# High cure rates and tolerability of artesunate–amodiaquine and dihydroartemisinin–piperaquine for the treatment of uncomplicated falciparum malaria in Kibaha and Kigoma, Tanzania

**DOI:** 10.1186/s12936-019-2740-z

**Published:** 2019-03-25

**Authors:** Celine I. Mandara, Filbert Francis, Mercy G. Chiduo, Billy Ngasala, Renata Mandike, Sigsbert Mkude, Frank Chacky, Fabrizio Molteni, Ritha Njau, Ally Mohamed, Marian Warsame, Deus S. Ishengoma

**Affiliations:** 1 National Institute for Medical Research, Tanga Research Centre, Tanga, Tanzania; 2 Kilimanjaro Christian Medical University College, Moshi, Tanzania; 3 Muhimbili University of Health and Allied Sciences, Dar es Salaam, Tanzania; 4 National Malaria Control Programme, Dar es Salaam, Tanzania; 5 Swiss Tropical and Public Health Institute, Dar es Salaam, Tanzania; 6 World Health Organization Country Office, Dar es Salaam, Tanzania; 7 Global Malaria Programme, World Health Organization, 20 Avenue Appia, 1211 Geneva 27, Switzerland; 8 Present Address: Gothenburg University, Gothenburg, Sweden

**Keywords:** Efficacy, Safety, Artesunate–amodiaquine, Dihydroartemisinin–piperaquine, *Plasmodium falciparum*, Tanzania

## Abstract

**Background:**

The Tanzanian National Malaria Control Programme (NMCP) and its partners have been implementing regular therapeutic efficacy studies (TES) to monitor the performance of different drugs used or with potential use in Tanzania. However, most of the recent TES focused on artemether–lumefantrine, which is the first-line anti-malarial for the treatment of uncomplicated falciparum malaria. Data on the performance of other artemisinin-based combinations is urgently needed to support timely review and changes of treatment guidelines in case of drug resistance to the current regimen. This study was conducted at two NMCP sentinel sites (Kibaha, Pwani and Ujiji, Kigoma) to assess the efficacy and safety of artesunate–amodiaquine (ASAQ) and dihydroartemisinin–piperaquine (DP), which are the current alternative artemisinin-based combinations in Tanzania.

**Methods:**

This was a single-arm prospective evaluation of the clinical and parasitological responses of ASAQ and DP for directly observed treatment of uncomplicated falciparum malaria. Children aged 6 months to 10 years and meeting the inclusion criteria were enrolled and treated with either ASAQ or DP. In each site, patients were enrolled sequentially; thus, enrolment of patients for the assessment of one artemisinin-based combination was completed before patients were recruited for assessment of the second drugs. Follow-up was done for 28 or 42 days for ASAQ and DP, respectively. The primary outcome was PCR corrected cure rates while the secondary outcome was occurrence of adverse events (AEs) or serious adverse events (SAEs).

**Results:**

Of the 724 patients screened at both sites, 333 (46.0%) were enrolled and 326 (97.9%) either completed the 28/42 days of follow-up, or attained any of the treatment outcomes. PCR uncorrected adequate clinical and parasitological response (ACPR) for DP on day 42 was 98.8% and 75.9% at Kibaha and Ujiji, respectively. After PCR correction, DP’s ACPR was 100% at both sites. For ASAQ, no parasite recurrence occurred giving 100% ACPR on day 28. Only one patient in the DP arm (1.1%) from Ujiji had parasites on day 3. Of the patients recruited (n = 333), 175 (52.6%) had AEs with 223 episodes (at both sites) in the two treatment groups. There was no SAE and the commonly reported AE episodes (with > 5%) included, cough, running nose, abdominal pain, diarrhoea and fever.

**Conclusion:**

Both artemisinin-based combinations had high cure rates with PCR corrected ACPR of 100%. The two drugs had adequate safety with no SAE and all AEs were mild, and not associated with the anti-malarials. Continued TES is critical to monitor the performance of nationally recommended artemisinin-based combination therapy and supporting evidence-based review of malaria treatment policies.

*Trial registration* This study is registered at ClinicalTrials.gov, No. NCT03431714

## Background

A substantial decline in malaria incidence, over 20% between 2010 and 2015, was observed in the past decade [1]. However, the most recent reports showed an increase in malaria incidence in 2016 and 2017, which might reverse the achievements attained in the past two decades [2, 3]. Malaria is still responsible for more than 435,000 deaths and over 219 million cases, with > 90% of the deaths and cases from sub-Saharan Africa (majority being under-fives and pregnant women) [3]. Despite a significant decline of malaria morbidity and mortality between 2000 and 2015 (> 18 million cases and > 100,000 deaths in 2000s) [4], Tanzania is still among the 10 high burden countries in Africa, with about 5.8 million cases and < 4000 deaths reported in 2017 [3].

Improved case management which involves early diagnosis (by microscopy or rapid diagnostic tests) and prompt treatment with effective anti-malarials is one of the current effective strategies to fight malaria [3]. Artemisinin-based combination therapy (ACT) recommended by the World Health Organization (WHO) is effective for the treatment of uncomplicated falciparum malaria and replaced monotherapies due to widespread resistance to previously used drugs [5]. Effective case management using ACT is believed to have significantly contributed to the recent reduction in malaria burden [1, 3]. Currently, the WHO recommends five artemisinin-based combinations for the treatment of uncomplicated falciparum malaria and these include artemether–lumefantrine (AL), artesunate–amodiaquine (ASAQ), artesunate-mefloquine (AS + MQ), dihydroartemisinin–piperaquine (DP) and artesunate-sulfadoxine/pyrimethamine (AS + SP) [6]. Pyronaridine–artesunate is another artemisinin-based combination which has been shown to have high therapeutic efficacy and safety for the treatment of uncomplicated malaria caused by *Plasmodium falciparum* and other species, and it will potentially offer an additional effective anti-malarial drug on top of the current ACT formulations [7–9]. Of these, AL is the commonly used ACT as the first-line anti-malarial for the treatment of uncomplicated malaria in most of malaria endemic countries, especially in the WHO African region followed by ASAQ [10]. Most of these artemisinin-based combinations still have high cure rates particularly in Africa [14–28], despite recent reports of artemisinin resistance and treatment failure with ACT in Southeast Asia (SEA) [11–16].

Due to high level of parasite resistance to SP and following WHO recommendations, Tanzania introduced AL as first-line drug for the treatment of uncomplicated malaria in 2006 [17]. Studies conducted in Tanzania before and after changes of the malaria treatment guidelines showed that AL is safe and efficacious (PCR corrected cure rate of > 95% by D28) [18–21]. However, confirmation of *P. falciparum* resistance to artemisinins [11, 13, 14] and other key partner drugs including piperaquine [22] in SEA, which was the epicentre for the evolution and spread of resistance to all important classes of anti-malarials, indicates that resistance to the currently recommended anti-malarial medicines could follow a similar mechanism and dispersal pattern [23]. Thus, the WHO recommends that regular surveillance to monitor efficacy and safety of ACT should be undertaken (biennial) by all malaria endemic countries in order to ensure optimum case management and facilitate early detection of emergence of artemisinin and partner drug resistance [24].

The Tanzania National Malaria Control Programme (NMCP), together with its partners, has been and are continuously implementing therapeutic efficacy studies (TES) to monitor the efficacy and safety of different anti-malarials including ACT that are being used or with potential future use in the country. The studies are conducted at eight sentinel sites located in regions with different malaria transmission intensities and also covering border areas, with high potentials of introducing parasites from neighbouring countries [25, 26]. These and previous studies which tested different anti-malarials in Tanzania [18], provided important evidence of the efficacy and safety of anti-malarials, and the data generated was used to support changes of malaria treatment guidelines to replace chloroquine with SP in 2001 [27] and AL to replace SP in 2006 [17]. Most of the recent studies focused on AL, which is the first line anti-malarial for the treatment of uncomplicated malaria in mainland Tanzania ([20], and Ishengoma et al. pers. commun.). However, data on the performance of other ACT is scanty [19, 20] and urgently needed to support timely review and changes of treatment guidelines in case of emergence of drug resistance to the current regimen. With confirmed resistance to artemisinins [11, 13, 14] and piperaquine in SEA [22], it is critical to ensure that studies to assess the efficacy and safety of other ACT are conducted in Tanzania. The current study was conducted at two NMCP sentinel sites to assess the efficacy and safety of ASAQ and DP for the treatment of uncomplicated malaria. Of these, ASAQ is the first-line anti-malarial in Zanzibar, and DP was recently introduced in Tanzania as an alternative ACT for the treatment of uncomplicated falciparum malaria, in order to improve case management when indicated or in case AL is out of stock [28].

## Methods

### Study sites

**Fig. 1:**
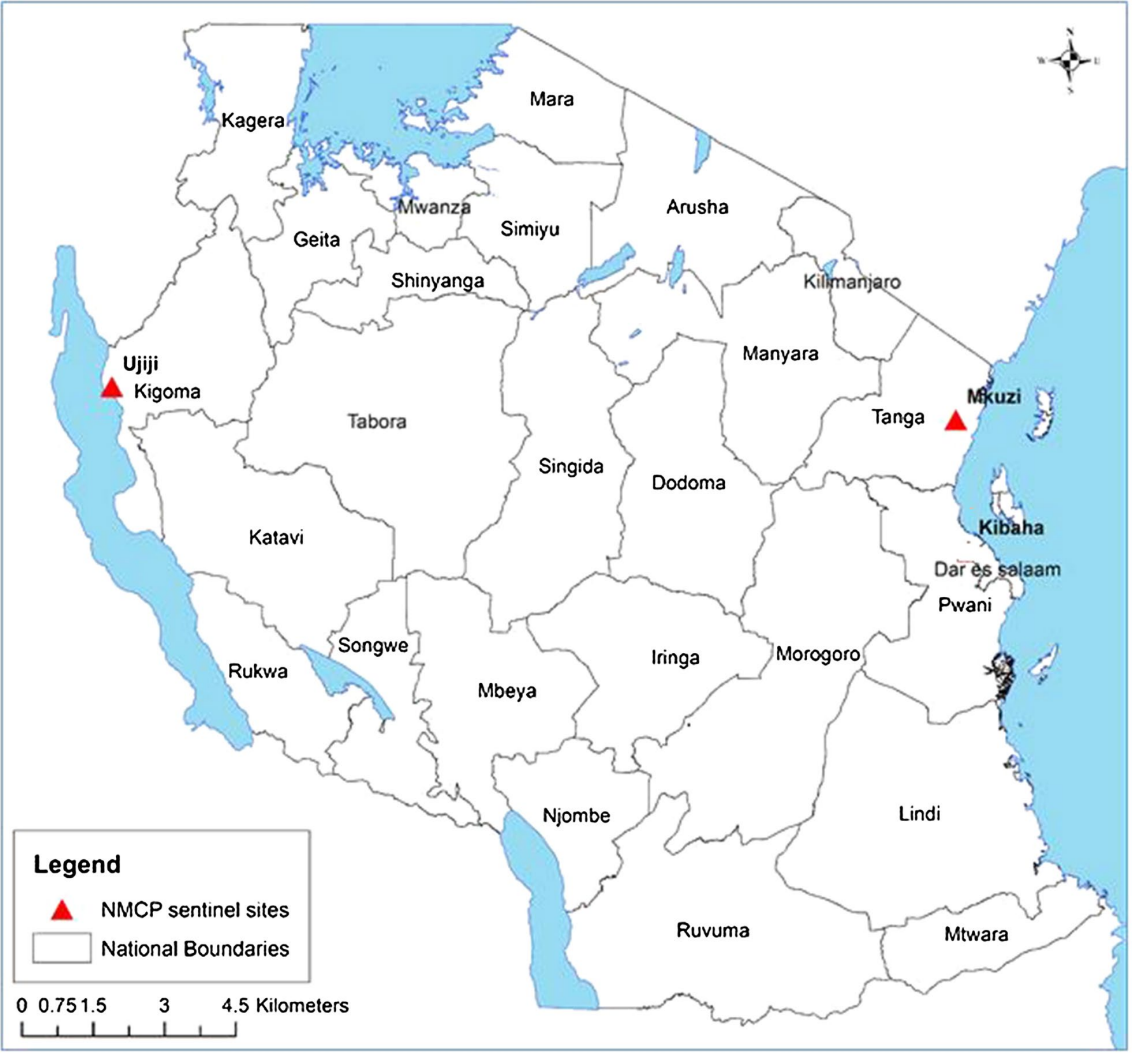
Map of Tanzania showing the two NMCP sentinel sites marked with red triangles

This study was carried out between July and December 2017 at Kibaha and Ujiji sites, which are among the 8 NMCP sentinel sites for monitoring anti-malarial efficacy in Tanzania [25, 26] (Fig. 1). The study was conducted at Magindu dispensary in Kibaha district in Coastal (Pwani) region, located about 100 km west of Dar es Salaam. Kibaha is currently characterized as a low malaria transmission site with prevalence among under-fives ranging from 5 to 10% in 2017 [29].

The second study site was Ujiji health centre which is located in the district of Kigoma urban in Kigoma region, north-western Tanzania. Despite high variability, the entire region of Kigoma is considered to be an area of high burden of malaria with parasite prevalence among under-fives ranging from 26% between 2012 and 2016 [30–32] to more than 43% in 2017 [29] and Chiduo et al. (pers. commun.). Further description of Ujiji site and Kigoma urban district was provided elsewhere [33].

### Study design and target population

This was a single-arm prospective study that assessed the therapeutic efficacy and safety of ASAQ and DP for the treatment of uncomplicated falciparum malaria. Children aged between 6 months to 10 years and attending the outpatients departments (OPDs) of the two health facilities were screened for possible inclusion in the study. Patients were first enrolled in the DP group between July and September 2017, until the sample size was attained before enrolment for ASAQ was initiated (September to November 2017). Enrolled patients were followed-up for 28 (ASAQ) or 42 days (DP) as per the WHO protocol of 2009 [24].

### Sample size estimation

The sample size was determined based on WHO standard protocol [24], with the assumption that 5% of the patients were likely to have a treatment failure after treatment with either of the two ACT. At a confidence level of 95% and an estimated precision of 5%; a minimum sample size was 73 patients in each treatment group at each of the two sites of Kibaha and Ujiji. The sample size was increased by 20% to allow for loss to follow-up and withdrawals during the 28 or 42-day follow-up for ASAQ and DP, respectively. The final sample size was 88 patients per drug per site and 352 for both drugs.

### Screening and recruitment of study participants

Children presenting to OPDs with age ranging between 6 months to 10 years and fever at presentation (axillary temperature ≥ 37.5 °C) or reported history of fever in the last 24 h were screened for possible enrolment, as previously described [33]. Recruitment of study participants at both sites was done starting with DP first followed with ASAQ. Once the sample size for DP was reached enrolment for ASAQ started. Since the study was undertaken in areas that have transitioned from high to moderate/low malaria transmission, older children (up to 10 years) were also enrolled and parasitaemia was adjusted to include those with 250–200,000 asexual parasites per microlitre of blood. Other inclusion and exclusion criteria were as per WHO protocol of 2009 [24], and as previously described [18–20].

### Laboratory examination

Laboratory screening involved a finger prick to obtain a blood sample for quick detection of malaria parasites using rapid diagnostic tests (RDTs) and collection of thick and thin blood smears for microscopy [33]. For patients with positive RDT results, two blood slides were collected and one of the slides was stained with 10% Giemsa for 10–15 min and examined by microscopy to detect presence of malaria parasites and the level of parasitaemia. The second blood slide was stained with 3% Giemsa for 30–45 min and used to determine the actual parasite density, species and presence of gametocytes. Detection of malaria parasites, parasite count and quality control of blood smears were undertaken as previously described [18, 19].

From each patient, dried blood spots (DBS) on filter papers (Whatmann No. 3, GE Healthcare Life Sciences, PA, USA) were collected for PCR genotyping to distinguish recrudescent from new infections. Extraction of parasite DNA from DBS was done at the laboratory in Tanga using QIAamp DNA blood mini kit (Qiagen GmbH, Hilden, Germany) according to the manufacturer’s instructions. Genotyping of paired samples (day 0 and parasites collected on or after day 14) was done by analysis of the highly polymorphic loci of merozoite surface proteins 1 and 2 (*msp1* and *msp2*), and glutamate rich protein (*glurp*) genes to distinguish true recrudescence from re-infection as previously described [24, 34].

### Treatment and follow-up

Patients enrolled in the study were treated with either ASAQ (Winthrop^®^, Sanofi Aventis, Morocco) or DP (Duo-Cortecxin^®^, Holley-Cotec Pharmaceuticals, China) obtained from WHO. The co-formulated ASAQ tablets contained 25 mg or 67.5 mg of artesunate and 50 mg or 135 mg amodiaquine in a tablet. DP was also a co-formulated regimen with tablets containing 40 mg and 320 mg of dihydroartemisinin and piperaquine, respectively. The drugs were administered according to the recommended doses based on body weight of patients. For ASAQ (25/67.5 mg), 1 tablet was given to children weighing < 10 kg; and for the 50/135 mg tablets, 1 tablet was given to those with 10–20 kg; 2 tablets to children with 21–30 kg and 3 tablets to children weighing > 30 kg. DP (40/320 mg) was administered with a quarter, half, a full tablet or 2 tablets given to patients weighing 5 to < 7 kg, 7 to  < 13 kg, 13 to  < 24 kg and 24 to  < 36 kg, respectively. A full course of either ASAQ or DP consisted of 3 doses given once daily after every 24 h. Patients were observed for 30 min to ensure that they did not vomit the study drugs. When vomiting occurred, a repeat dose was given after vomiting stopped. Any patient who persistently vomited the study medication was withdrawn and treated with intravenous quinine or intramuscular artesunate according to the national guidelines for management of severe malaria [17]. Paracetamol was given to all patients with body temperature greater than or equal to 38 °C. All doses of the study drugs were administered orally under direct observation of a study nurse.

Scheduled follow-up visits were done on days 1, 2, 3, 7, 14, 21 and 28 for ASAQ or with 2 extra visits on 35 day and 42 for DP; or at any other time (unscheduled visits) when patients felt unwell. Parents/guardians were asked to bring their children to the clinic at any time when they felt unwell without waiting for scheduled visits or taking them to other health facilities for medical attention. All patients who failed to turn up for their scheduled visits by mid-day were followed-up at their respective homes by a member of the study team and asked to come to the health centre for their visits. Patients who travelled away from the centre and could not be traced, were classified as lost and withdrawn from the study. During the visits, both clinical and parasitological assessments were performed; and follow-up samples (blood slides and DBS) were also collected.

### Safety assessment

Both passive and active methods were used to assess the safety of the two drugs through interviews with parents/guardian to capture and report adverse events (AEs) or serious adverse events (SAEs). Parents/guardian were interviewed at each visit and asked to report any occurrence and nature of AE or SAE that occurred at home between follow-up visits. At the study facilities, clinical examination and/or laboratory tests were also used to determine and capture AEs and SAEs. The captured events were recorded on case report forms for each follow-up visit. An AE or SAE were defined and classified according to WHO protocol [24]. Reporting procedures for any SAE included submission of a written report by the principal investigator to the sponsor (the National Institute for Medical Research—NIMR), NMCP and the Tanzanian Medical Research Coordinating Committee (MRCC) of NIMR (which is the national ethics review board in Tanzania). Reporting of an SAE was done within 24 h of occurrence regardless of whether the principal investigator considered the event to be related to the investigated drug or not. Patients with AEs or SAEs were thoroughly assessed and managed accordingly, and the events were also assessed to determine their association with the study drugs.

### Outcome classification

The primary end point was parasitological cure on day 28 for ASAQ and day 42 for DP as per WHO protocol of 2009 [24], while secondary end points included parasitaemia on day 3 and occurrence of AEs/SAEs. The primary treatment outcomes were classified as early treatment failure (ETF), late clinical failure (LCF), late parasitological failure (LPF), and adequate clinical and parasitological response (ACPR) before and after PCR correction [24]. Rescue treatment for recurrent infections identified during follow-up was done using artemether–lumefantrine while patients with severe malaria were managed with intravenous quinine or intramuscular artesunate.

### Ethical considerations

The protocol was reviewed and approved by the MRCC of NIMR and permission to conduct the study at the health facilities was sought in writing from the relevant regional and district medical authorities. Oral and written informed consent was obtained from parents or guardians of all eligible patients before their children were screened for possible inclusion into the study. Information about the study protocol, inclusion criteria, follow-up schedule, and benefits and risks of participating in the study was provided to parents/guardians during the consenting process. The study is registered at ClinicalTrials.gov, No. NCT03431714.

### Data management and analysis

Single entry was concurrently performed at the study sites during data collection while the second entry was done by another data entry clerk after the end of fieldwork. The data were entered into a Microsoft Access database accessible online via internet, and was later validated, cleaned and analysed using STATA for Windows, version 13 (STATA Corporation, TX-USA). The data were also transferred to the WHO Excel software programme [35], for automatic analysis of treatment outcomes. Descriptive statistics such as percentages, mean, median, standard deviation, and range were reported as appropriate. Treatment outcomes were analysed based on per protocol method and Kaplan–Meier analysis and reported as uncorrected and PCR corrected cure rates. Baseline characteristics, primary and secondary outcomes were compared between the two sites for each drug. Continuous variables such as log_10_ transformed parasite density (at enrolment) and age of patients from the two sites were compared using *t* test (for normally distributed data) or Mann–Whitney U test (a non-parametric test for non-normally distributed data). Distinguishing recrudescent from new infections was done using *msp2* followed by *glurp* and *msp1* based on the WHO protocol [24, 34]. Inconclusive results were reported as non-determined and excluded from the analysis of treatment outcomes. For all statistical tests, p-value of < 0.05 was considered to be significant.

## Results

### Baseline characteristics

**Fig. 2:**
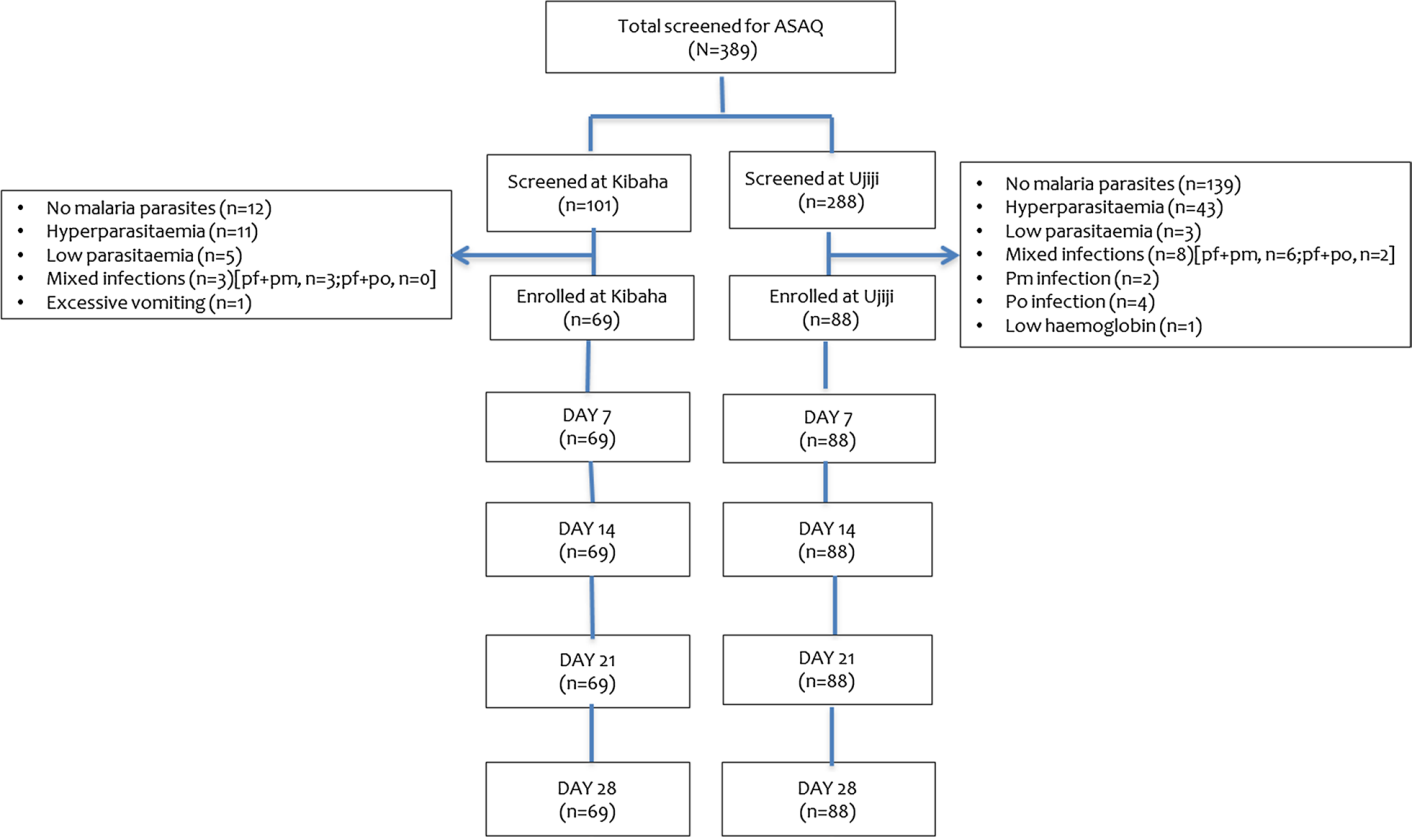
Trial profile for ASAQ showing the flow of patients during screening, enrolment and follow-up

**Fig. 3:**
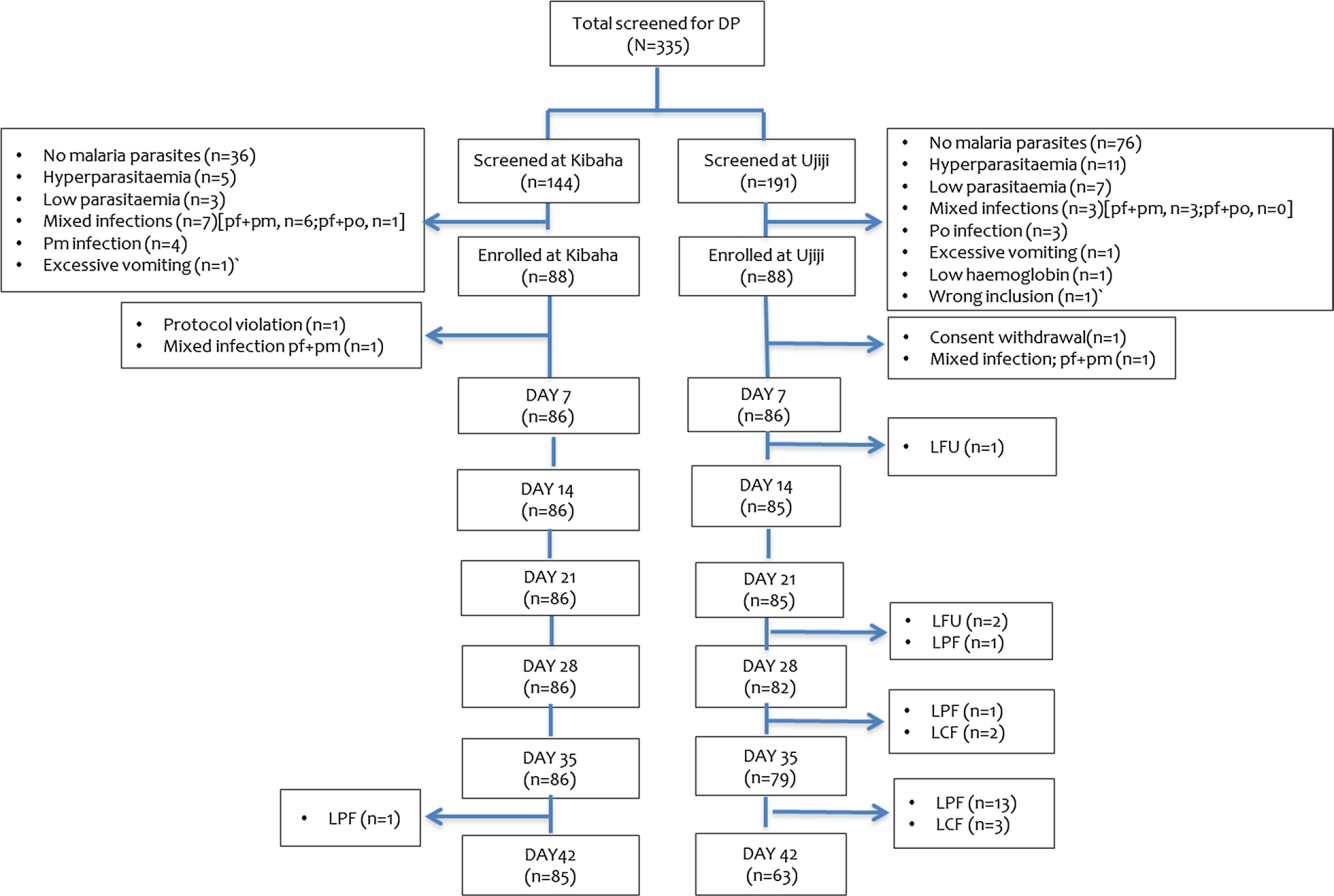
Trial profile for DP showing the flow of patients during screening, enrolment and follow-up

A total of 724 patients were screened at both sites, 333 (46.0%) were enrolled into the study and 326 (97.9%) completed the follow-up visits or had an assigned treatment outcome (Figs. 2 and 3). There was no significant difference in age (p = 0.097), axillary temperature (p = 0.216) and sex (p = 0.946) of patients recruited at the 2 sites (Table 1). Children recruited at Kibaha had significantly higher weight (p = 0.003) and height (p < 0.001) compared to those from Ujiji. In addition, geometric mean parasite density was significantly higher at Ujiji than Kibaha (p = 0.013) (Table 1).

**Table 1:** Baseline characteristics of children enrolled at Kibaha and Ujiji

Variable	Study sites				Overall
	Kibaha		Ujiji		
	DP	ASAQ	DP	ASAQ	
Screened	144	101	191	288	724
Enrolled	88 (61.1)	69 (68.3)	88 (46.1)	88 (30.6)	333 (46.0)
Age in years, mean (SD)	5.5 (2.8)	5.0 (2.7)	4.8 (2.6)	4.7 (2.8)	5.0 (2.7)
Gender (male), n (%)	54 (61.4)	34 (49.3)	45 (51.1)	53 (63.2)	186 (55.7)
Weight (kg), median (IQR)**	17 (13.3–21)	15 (12.5–22)	14 (12–18)	14.6 (11–18.5)	15 (12–19)
Height in cm, median (IQR)***	109 (93–123)	104 (92.5–121)	101 (83–112)	101 (86–115)	104 (90–119)
Body temp (°C) ± mean (SD)	37.9 (1.2)	37.8 (1.1)	38.2 (1.3)	38.0 (1.4)	38.0 (1.2)
Parasitaemia-GMPD (asexual pf/µl) 95% CI*	24,974 (18,822–33,136)	24,441 (16,166–36,954)	36,726 (27,481–49,082)	36,137 (28,809–45,329)	30,357 (26,157–35,231)

°C, degree Celsius; SD, standard deviation; GMPD, Geometric mean parasite density; pf, *Plasmodium falciparum*; 95% CI, 95% confidence interval; n, number of patients; IQR, Inter quartile range; µl, microlitre; ETF, early treatment failure; LCF, late clinical failure; LPF, late parasitological failure; ACPR, adequate clinical and parasitological response; PP, number of patients involved in the per protocol analysis; LFU, lost to follow-up; WD, withdrawn; n, number of episodes

* p = 0.013, ** p = 0.003, *** p < 0.001

### Treatment outcomes

Three patients (0.9%) were lost to follow-up at both sites while four (1.2%) (two from each site in the DP group) were withdrawn from the study for different reasons (Fig. 2 and Tables 2, 3). A total of 326 (97.9%) patients attained the study outcomes and were used in per protocol analysis, while in the Kaplan–Meier analysis; patients lost to follow-up and those withdrawn were included in the analysis until the last day seen. Before PCR correction (on day 42), 1 (1.2%) and 15 (18.1%) patient in the DP group at Kibaha and Ujiji, respectively, had LPF while 5 (6.0%) patients in the DP group from Ujiji site had LCF. For DP, PCR uncorrected ACPR of 98.8% (85/86) was reported at Kibaha on day 28 while it was 100% at Ujiji (Table 2). On day 42, PCR uncorrected ACPR of DP was 98.8 (85/86) and 75.9% (63/83) at Kibaha and Ujiji, respectively (Table 3). After PCR correction, all recurrent infections in the DP group from both sites were new infections and PCR corrected ACPR was 100.0% (Tables 2 and 3). For ASAQ, there was no treatment failure in both sites and the PCR uncorrected ACPR on day 28 was 100.0% (Table 2). Only one patient in the DP arm (1.1%) from Ujiji had parasites on day 3 (Tables 2 and 3).

**Table 2:** Treatment outcomes before and after PCR genotyping on day 28

Item	Kibaha		Ujiji		Total
Outcome	DP (n = 88)	ASAQ (n = 69)	DP (n = 88)	ASAQ (n = 88)	N = 333
PCR uncorrected					
Day 3 parasitaemia	0 (0.0%)	0 (0.0%)	1 (1.1%)	0 (0.0%)	1 (0.3%)
ETF	0 (0.0%)	0 (0.0%)	0 (0.0%)	0 (0.0%)	0 (0.0%)
LPF	1 (1.2%)	0 (0)	0 (0.0%)	0 (0)	1 (0.3%)
LCF	0 (0)	0 (0)	0 (0.0%)	0 (0)	0 (0.0%)
ACPR	85 (98.8%)	69 (100%)	83 (100%)	88 (100%)	325 (99.6%)
Total PP	86	69	83	88	326
LFU	0 (0%)	0 (0%)	3 (3.4%)	0 (0%)	3 (0.9%)
WD	2 (2.3%)	0 (0%)	2 (2.3%)	0 (0%)	4 (1.2%)
PCR corrected					
ETF	0 (0.0%)	0 (0.0%)	0 (0.0%)	0 (0.0%)	0 (0.0%)
LPF	0 (0.0%)	0 (0.0%)	0 (0.0%)	0 (0.0%)	0 (0.0%)
LCF	0 (0.0%)	0 (0.0%)	0 (0.0%)	0 (0.0%)	0 (0.0%)
ACPR	85 (100%)	69 (100%)	83 (100%)	88 (100%)	325 (100%)
Total PP	85	69	83	88	325
LFU	0 (0.0%)	0 (0%)	3 (3.4%)	0 (0.0%)	3 (0.9%)
WD	2 (2.3%)	0 (0.0%)	2 (2.3%)	0 (0.0%)	4 (1.2%)

**Table 3:** Treatment outcomes for DP before and after PCR genotyping on day 42

Item	Kibaha	Ujiji	Total
Outcome	DP (n = 88)	DP (n = 88)	N = 176
PCR uncorrected			
Day 3 parasitaemia	0 (0.0%)	1 (1.1%)	1 (0.6%)
ETF	0 (0.0%)	0 (0.0%)	0 (0.0%)
LPF	1 (1.2%)	15 (18.1%)	16 (9.5%)
LCF	0 (0)	5 (6.0%)	5 (3.0%)
ACPR	85 (98.8%)	63 (75.9%)	148 (87.6%)
Total PP	86	83	169
LFU	0 (0%)	3 (3.4%)	3 (1.7%)
WD	2 (2.3%)	2 (2.3%)	4 (2.3%)
PCR corrected			
ETF	0 (0.0%)	0 (0.0%)	0 (0.0%)
LPF	0 (0.0%)	0 (0.0%)	0 (0.0%)
LCF	0 (0.0%)	0 (0.0%)	0 (0.0%)
ACPR	85 (100%)	63 (100%)	148 (100%)
Total PP	85	63	148
LFU	0 (0.0%)	3 (3.4%)	3 (1.7%)
WD	3 (3.4%)	22 (25.0%)	25 (7.5%)

### Safety outcomes

Among the patients recruited in this study (n = 333), 175 (52.6%) had one or more events of AEs, with 223 episodes of AEs at both sites. The commonly reported AE episodes included cough (39.0%), running nose (14.8%), abdominal pain (7.2%), diarrhoea (7.2%), fever (6.7%), vomiting (4.0%), skin itching (3.1%), painful micturition (2.7%), painful ear (2.7%), difficulty in breathing (2.2%) and others (Table 4). Other AEs (n = 23) included 4 events each of headache, painful swallowing and mouth sore; three events were reported for loss of appetite and painful eyes; and one event each of chicken pox, common cold, anaemia, blood in stool and visible worms. No case of SAE was reported at the two sites and all AEs were managed accordingly.

**Table 4:** Episodes of adverse events reported at Kibaha and Ujiji

AEs	Kibaha		Ujiji		Total (n = 223)
	DP (n = 73)	ASAQ (n = 38)	DP (n = 62)	ASAQ (n = 50)	
Cough	37 (50.7)	19 (50.0)	15 (24.2)	16 (32.0)	87 (39.0)
Running nose	15 (20.5)	3 (7.9)	7 (11.3)	8 (16.0)	33 (14.8)
Abdominal pain	4 (5.5)	2 (5.3)	6 (9.7)	4 (8.0)	16 (7.2)
Diarrhoea	2 (2.7)	1 (2.6)	7 (11.3)	6 (12.0)	16 (7.2)
Fever	3 (4.1)	1 (2.6)	8 (12.9)	3 (6.0)	15 (6.7)
Vomiting	3 (4.1)	0 (0)	3 (4.8)	3 (6.0)	9 (4.0)
Skin itching	3 (4.1)	1 (2.6)	3 (4.8)	0 (0)	7 (3.1)
Painful micturition	0 (0)	1 (2.6)	4 (6.5)	1 (2.0)	6 (2.7)
Painful ear	1 (1.4)	1 (2.6)	2 (3.2)	2 (4.0)	6 (2.7)
Difficulty in breathing	0 (0)	0 (0)	1 (1.6)	4 (8.0)	5 (2.2)
Others	5 (6.8)	9 (23.7)	6 (9.7)	3 (6.0)	23 (10.3%)

n = number of episodes

## Discussion

This study was conducted to assess efficacy and safety of ASAQ and DP which are alternative ACT in Tanzania in order to provide data that will potentially support the review process and formulation of new treatment guidelines in case of resistance to the current anti-malarials. The findings showed high efficacy of ASAQ and DP at both sites, where the cure rate of ASAQ (PCR uncorrected ACPR = 100%) was higher compared to what was reported in other studies done in both Mainland Tanzania and Zanzibar [36–38]. The cure rate of ASAQ was also higher compared to previous studies from other East African countries [39–44] and across Africa [45, 46]. These results could possibly be due to resumption of parasite sensitivity to amodiaquine after it was withdrawn in 2006 when the interim treatment guidelines of 2001 (which had SP as first-line and amodiaquine as the second-line anti-malarial) were changed to introduce ACT [17].

Previous studies conducted in Tanzania before and after the 2006 policy changes showed low PCR corrected cure rates among patients treated with ASAQ which ranged from 88 to 94% [18] and this was possibly due to high resistance to amodiaquine monotherapy [38, 47, 48]. Furthermore, studies conducted in Tanzania and other countries in SSA showed high treatment failure to amodiaquine which is a partner drug in the ASAQ combination [18, 39, 41, 43–46, 49]. Thus, withdrawal of amodiaquine monotherapy and introduction of AL could have resulted into restoration of amodiaquine sentistivity due to reduced drug pressure leading to high efficacy of ASAQ. However, studies have also showed that increased use of lumefantrine as a partner drug selects for parasites which are sensitive to both chloroquine and amodiaquine [23]. Further surveillance will be required to monitor the performance of ASAQ in areas such as Mainland Tanzania where drug pressure caused by amodiaquine is continuously reduced by use of AL.

According to WHO, the proportion of patients with parasitaemia on day 3 post-treatment (day 3 positivity rate) should be reported as an important indicator for identifying suspected artemisinin partial resistance in *P. falciparum* [50]. In this study, all patients but one (99.7%) cleared parasites on day 3. This is consistent with studies done in Tanzania [15–18] and other African countries [51–53], further suggesting that partial resistance to artemisinin has not emerged in Tanzania. The high PCR corrected cure rate for DP in this study supports earlier findings from Tanzania, whereby the PCR corrected cure rates ranging from 94.6 to 100% have been recently reported in Kyela, Muheza and Ujiji [19, 20]. Similarly, studies undertaken in the East African regions [51, 54–56] and across Africa [57, 58] have reported high cure rates among patients with uncomplicated malaria treated with DP.

Although the current study reported day 42 PCR corrected ACPR of 100%, a high rate of recurrent infection was also reported in the DP group at Ujiji and all occurred after day 28. PCR uncorrected ACPR of both drugs on day 28 was 100% and all recurrent infections in the DP group were confirmed to be new infections after PCR genotyping. This is a major concern for effective case management since previous studies involving other artemisinin-based combinations [18, 19] also reported higher rates of recurrent infections particularly in areas with high malaria transmission like Kigoma. In the previous studies, recurrent infections among patients treated with different artemisinin-based combinations (most of them were due to new infections) were attributed to high transmission of malaria in Kigoma and other sites where high prevalence among under-fives has been reported [29–32]. In Kigoma, a recent study also reported high density of *Anopheles funestus* (Chiduo et al., pers. commun.), which is a highly potent malaria vector, suggesting that more strategies and targeted malaria control interventions are urgently required to reduce the burden of malaria in this region. From the NMCP perspective, there is a need to ensure that preventive measures like insecticide-treated bed nets are made available and used by the population living in these areas.

This study also showed that the two drugs were well tolerated with minimal AEs and with no any SAE. Of the AEs, majority of the cases (39.0%) had episodes of cough and the rest had other mild symptoms. Studies conducted in Tanzania [18, 20] and elsewhere in Africa [58] reported similar safety profile of ASAQ and DP when the drugs were used for the treatment of uncomplicated falciparum malaria.

## Conclusion

This study showed that the two artemisinin-based combinations had high efficacy for the treatment of uncomplicated falciparum malaria with PCR corrected cure rate of 100% for both drugs. The drugs had adequate safety profile with no SAE and all the reported AEs were mild, and resolved on their own or after medical interventions. Further surveillance will be required to assess the efficacy and safety of these alternative forms of ACT in order to support regular review of malaria treatment guidelines in Tanzania.

## Abbreviations

ACPR: adequate clinical and parasitological response
ACT: artemisinin-based combination therapy
AE: adverse event
AL: artemether–lumefantrine
ASAQ: artesunate–amodiaquine
DBS: dried blood spots on filter papers
DP: dihydroartemisinin–piperaquine
ETF: early treatment failure
*glurp*: glutamate-rich protein gene
LCF: late clinical failure
LPF: late parasitological failure
MRCC: Medical Research Coordinating Committee
RDTs: rapid diagnostic tests for malaria
*msp1*: merozoite surface protein 1 gene
*msp2*: merozoite surface protein 2 gene
NIMR: National Institute for Medical Research
NMCP: National Malaria Control Programme
PCR: polymerase chain reaction
SAE: serious adverse event
TES: therapeutic efficacy studies
WHO: World Health Organization
